# Nonlinear Dynamics Analysis of Handgrip Strength Using the Poincaré Plot Method Through Video Processing Techniques

**DOI:** 10.3390/jfmk9040234

**Published:** 2024-11-13

**Authors:** Constantin Ciucurel, Elena Ioana Iconaru

**Affiliations:** Department of Medical Assistance and Physical Therapy, University Center of Pitesti, National University of Science and Technology Politehnica Bucharest, 110040 Pitesti, Romania; constantin.ciucurel@upb.ro

**Keywords:** hand grip strength, nonlinear dynamics, Poincaré plot, time series, motion capture techniques

## Abstract

Background/Objectives: The aim of this study was to analyze the nonlinear dynamics of handgrip strength (HGS) in young adults, focusing on hand dominance, by employing the Poincaré plot method to assess short- and long-term variability utilizing dynamometry and video motion capture during sustained isometric contractions. Methods: A cross-sectional exploratory study was conducted on 30 healthy subjects (mean age 21.6 ± 1.3 years, 13 males and 17 females), measuring HGS for both the dominant hand (DH) and nondominant hand (NDH) using a Saehan hydraulic dynamometer during 25-s sustained isometric contractions. A GoPro HERO11 Black camera recorded the dynamometer’s needle movements, and the video data were analyzed using Kinovea software. Angular values were converted to force using a calibration-based formula, and the Poincaré plot computed variability indices (short-term variability—SD_1_, long-term variability—SD_2_, ratio SD_1_/SD_2_, and area of the fitting ellipse) for each hand in relation to HGS and angular velocity (AV). Data analysis included descriptive and inferential statistics. Results: We demonstrated a strong correlation between mechanical and video measurements (*p* ≤ 0.001), confirming the reliability of the video method. The findings highlight the importance of nonlinear analysis in understanding neuromuscular function and fatigue, revealing significant correlations among HGS, AV, Poincaré indices, and fatigue levels in both hands (*p* ≤ 0.001). Increased maximum HGS and AV correlated with higher nonlinear variability in force production. Conclusions: This study confirms the reliability of the proposed video-based HGS assessment and demonstrates the effectiveness of Poincaré plot analysis for capturing nonlinear variability in HGS.

## 1. Introduction

Handgrip strength (HGS) is a fundamental measure of muscular strength and has widespread clinical applications, particularly in assessing overall health status, diagnosing neuromuscular diseases, and predicting future health outcomes. The measurement of HGS provides critical insights into an individual’s muscle function [1], which is essential in various medical fields including geriatrics, rehabilitation, and sports medicine. It serves as an indicator of functional ability and physical performance, making it a valuable tool in clinical settings for evaluating patient strength and predicting disability and mortality risks [2]. Additionally, HGS serves as a robust biomarker of biological age, with its decline being strongly correlated with the aging process [3,4].

Traditionally, HGS is assessed through an isometric contraction of the hand muscles, with the peak value recorded as the primary measure. However, the dynamic aspects of HGS, especially the nonlinear variations observed during repetitive testing or prolonged contractions, have been less extensively investigated. These variations are intrinsically linked to the concept of entropy [5]. Nonlinear variation in physiological data, such as HGS, reveals complex underlying dynamics that linear methods may fail to capture. Traditional linear analyses often overlook these complexities, whereas nonlinear analysis, such as the Poincaré plot method, offers a more comprehensive understanding of the physiological processes [6]. The Poincaré plot, a geometric tool for visualizing the variability of time series data, enables the assessment of both short-term and long-term fluctuations in different types of variables. By mapping successive pairs of data points onto a Cartesian coordinate system, it reveals dynamic patterns within the series. Originally developed for the time series analysis of signals, this method has also proven useful in kinesiological studies, providing critical insights into the complex movement patterns and biomechanics of the human body [7].

The method provides key descriptors such as SD_1_ and SD_2_, representing short-term and long-term variability, respectively, and their ratio (SD_1_/SD_2_), which indicates the relative balance between these variabilities, as well as the area of the fitting ellipse (AFE), which quantifies the overall variability [5,8]. The Poincaré plot, through descriptive indices, effectively captures subtle correlation patterns and dynamic variability in time series, offering insights beyond traditional analysis methods [9]. This nonlinear technique, which utilizes return maps of consecutive intervals, enhances the analysis by revealing complex underlying dynamics that are often missed by linear approaches [10]. Nonlinear methods provide a deeper understanding because they can capture complex non-additive relationships and patterns that linear approaches, which assume proportionality and constant rates of change, cannot.

Another advantage of the Poincaré plot is its ability to identify cyclic patterns and temporal dynamics between successive data points, which are often challenging to detect using conventional spectral analysis methods [11]. In biomechanical parameters, the Poincaré plot can visualize the relationship between consecutive measurements, enabling a detailed assessment of movement variations and patterns relevant for evaluating fluctuations in different physiological or pathological conditions [12]. This method appears attractive in HGS analysis, as it captures subtle fluctuations and cyclic patterns in hand strength that traditional methods often miss.

In recent years, advanced video processing techniques, enhanced by artificial intelligence, have become essential for biomechanical analyses in clinical patient monitoring. These techniques provide precise non-invasive assessments that significantly enhance and improve the diagnosis and evaluation of physiological parameters [13]. Specifically, in the case of HGS, video processing techniques have been underutilized, with limited references in the published literature. Most research has focused on other applications, such as automated assessments of repetitive hand activity [14] and video game-based systems for measuring muscle force in children [15]. However, these techniques hold the potential to offer detailed and dynamic evaluations of muscle force, thereby enhancing the accuracy and depth of clinical insights.

In this study, we propose a novel approach that integrates advanced video processing techniques for motion capture with nonlinear dynamics analysis to investigate the variability of HGS during sustained isometric contractions. This study aims to explore the nonlinear dynamics of HGS in a cohort of young adults, analyzing the data in the context of hand dominance. By applying the Poincaré plot method, we seek to quantify the variability in HGS, comparing the dominant hand (DH) and nondominant hand (NDH). An additional objective of this study is to conduct a correlational analysis between the maximal HGS during sustained isometric contractions and the nonlinear variability indices derived from the Poincaré plot (SD_1_, SD_2_, AFE, and SD_1_/SD_2_). This innovative approach not only enhances the understanding of muscular force dynamics but also introduces a novel methodology for capturing and analyzing HGS data through video processing techniques. Our findings could have significant implications for clinical assessments and interventions, providing deeper insights into the neuromuscular function and its variability.

## 2. Materials and Methods

### 2.1. Study Design

This research is a cross-sectional exploratory study aimed at analyzing the nonlinear dynamics of HGS through the Poincaré plot method using video processing techniques. The study was designed to investigate HGS among healthy young adults by capturing the angular movement of the dynamometer needle during isometric contractions. The primary objective was to evaluate the relationship between hand dominance and HGS variability, thereby enhancing our understanding of muscular performance in this population.

### 2.2. Study Population and Settings

The study was conducted on a sample of 30 healthy subjects aged 20 to 24 years (mean age 21.6 ± 1.3 years; 13 males and 17 females). The inclusion criteria for subjects were as follows: participants had to be in good health with no known neuromuscular or orthopedic conditions that could affect HGS and had a clearly defined DH as self-reported. Hand dominance was determined based on preferences in daily activities, with a particular emphasis on eating and writing [16]. As for anthropometric data, the participants had a mean height of 170.83 ± 9.76 cm and a mean weight of 68.43 ± 18.9 kg. Informed consent was obtained from each subject.

**Figure 1:**
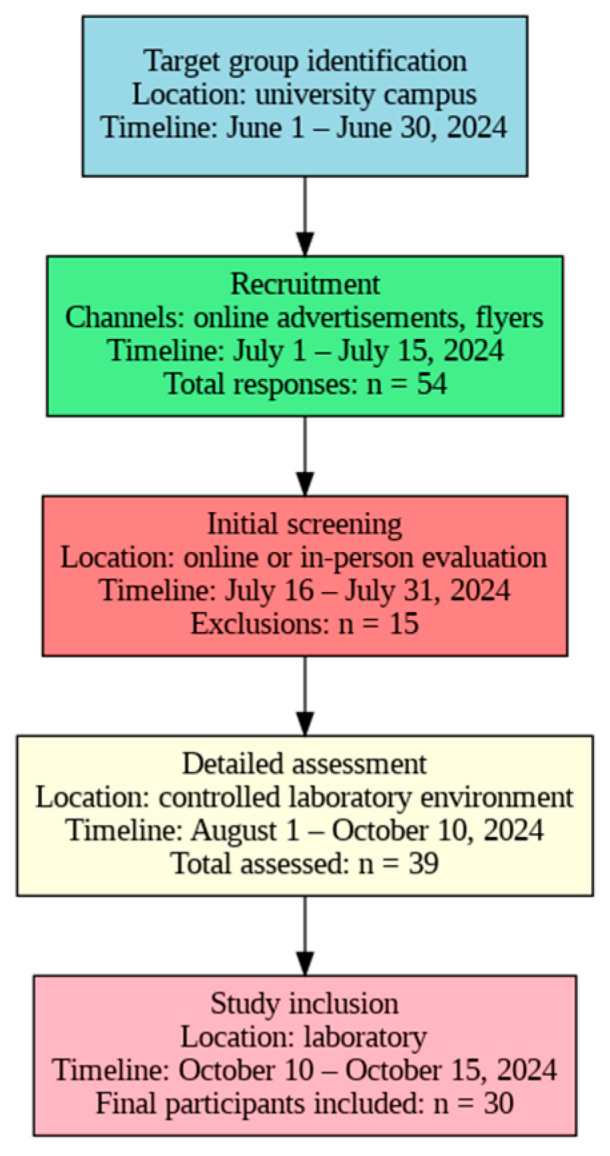
Flowchart of the recruitment process for participants in the study (n: number of subjects).

The study was conducted in a controlled laboratory environment to minimize distractions. All procedures were approved by the local ethics committee, ensuring adherence to ethical standards throughout the research process. This careful selection of participants and the controlled setting were essential for obtaining reliable data regarding HGS. The flowchart illustrating the recruitment process, along with the corresponding locations and timelines, is presented in Figure 1.

### 2.3. Study Size

To determine the sample size, we considered an anticipated effect size of 0.8, a significance level (α) of 0.05, and a statistical power of 0.8. Based on the power analysis conducted, these parameters indicated that a sample size of 30 subjects would be required to reliably detect the anticipated effect in our study [17].

### 2.4. Data Sources/Measurement

For each participant, HGS was measured for both hands alternately using a hydraulic hand dynamometer (Saehan model, MSD Europe bvba, Brussels, Belgium). The measurements were recorded separately for each hand and expressed in kilogram-force (kgf). Participants sat on a chair with their elbow flexed at 90° and their forearm resting on a table. The fixed part of the dynamometer was aligned with a marked line, and participants were instructed to keep their forearm in this position throughout the evaluation. The DH was tested first, followed by the ND, with a 5-min break between measurements. For each hand, participants were instructed to perform and maintain a maximal contraction at a continuous maximum level for 25 s. The recommendation was to maintain a static position of the upper limb as much as possible, with movements allowed only at the hand level, without moving the screen of the dynamometer.

This type of testing was based on international recommendations [18], but the protocol was adapted to facilitate video recording of the dynamometer screen from a fixed distance. To achieve this, we captured the oscillations of the dynamometer needle using a high-resolution GoPro HERO11 Black camera (GoPro, Inc., San Mateo, CA, USA). The camera was mounted on a tripod, positioned at a fixed horizontal distance of 14 cm from the dynamometer screen, and a height of 96 cm above the ground. Participants were instructed to keep the dynamometer as steady as possible, with its screen parallel to the camera’s, throughout the measurements. The center of the screen was aligned at the same height as the camera lens to ensure accurate video capture. This helps maintain consistency in the measurements and avoid distortion or parallax errors in the analysis of the needle’s movements. The recording was conducted with standard settings of 4 K video at 30 frames per second (fps), using a linear lens.

The recorded video was then analyzed using Kinovea software, version 2023.1.2. (https://www.kinovea.org) (accessed on 1 October 2024), a freely available 2D motion analysis tool that is well-regarded for its reliability and accuracy in research applications, including sports and clinical settings [19,20]. To measure the angle between the zero line and the dynamometer needle, the following steps were taken using the Kinovea software, version 2023.1.2., on the recorded video: we first established a plane calibration, followed by compensating for lens distortion. Next, we tracked the angle throughout the 25-s interval, with manual corrections made to the automatic tracking when necessary, and finally exported the angular kinematics for further analysis. The software extracts time-series data at 33.33 ms intervals, recording the variations in the angle. The variables exported from Kinovea in .csv format include the angle between the zero line and the dynamometer needle (deg) and angular velocity (AV, deg/s). We then transformed the angular values of the needle into force measurements expressed in kilograms-force (kgf) using the following formula:

Force = angle × 90/298(1)

The value of 298 degrees in the formula, determined through camera calibration, represents the total angular range of the dynamometer needle, from 0 kgf to 90 kgf, thereby allowing the conversion of angular displacement into measurable force output. Considering the linear relationship between angle and force, the AV can be regarded as analogous to the rate of change in force.

The recordings provided data series for both the DH and NDH. For each recorded variable, we applied the Poincaré plot model for mathematical data processing, using standard formulas for the parameters SD_1_, SD_2_, SD_1_/SD_2_, and AFE [21,22,23,24], as follows:

(2) $${\mathrm{SD}}_{1}=\frac{\sqrt{2}}{2}*SD\left(x_{n}-x_{n+1}\right)$$

(3) $${\mathrm{SD}}_{2}=\sqrt{2{SD\left(x_{n}\right)}^{2}-\frac{1}{2}{SD\left(x_{n}-x_{n+1}\right)}^{2}}$$

(4) $$\mathrm{AFE}=\pi *{SD}_{1}*{SD}_{2}$$

In these formulas, SD(x_n_ − x_n+1_) is the standard deviation (SD) of the differences x_n_ − x_n+1_ from the data series, while SD(x_n_) represents the SD of x_n_.

Practically, SD_1_ measures short-term variability by capturing the dispersion of points perpendicular to the line of identity (45-degree line) in a Poincaré plot, while SD_2_ measures long-term variability by capturing the dispersion along this line [25]. AFE is the area of the ellipse fitted to the Poincaré plot, representing the overall variability and correlation structure of the data [26]. In addition, we also calculated the SD_1_/SD_2_ ratio, which measures the balance between short-term and long-term variability in the data [27]. A higher ratio indicates greater short-term variability, while a lower ratio suggests that long-term trends dominate. This ratio helps distinguish between random noise and persistent patterns in a time series.

### 2.5. Variables and Statistical Methods

The data series for HGS, grouped by hand dominance (DH and NDH), were statistically processed using IBM SPSS 26.0 software (IBM Corp., Armonk, NY, USA) [28]. Initially, descriptive statistics, including the mean, SD, maximum, and minimum values for HGS and AV, were calculated for each subject’s DH and NDH. These values were derived from data captured at 33.33 ms intervals over a 25-s isometric contraction period for each subject, for both the DH and NDH tests. Furthermore, the Poincaré plot parameters (SD_1_, SD_2_, SD_1_/SD_2_, and AFE) were computed for each subject’s hand in relation to HGS and AV. Subsequently, the group-level means and SD for the Poincaré plot parameters were calculated, considering hand laterality. The Shapiro–Wilk test was used to assess the normality of all variables at the group level. For variables that did not exhibit a normal distribution, the Mann–Whitney U test was utilized to assess the statistical significance of differences in ranks between groups, with U and z values calculated for comparison. To assess the magnitude of these differences, the effect size coefficient r was calculated using the following formula:

(5) $$r=\frac{z}{\sqrt{n}}$$

where n represents the sample size.

Effect sizes were interpreted as follows: r > 0.5, indicating a large effect, r = 0.3–0.5 a medium effect, and r < 0.3 a small effect [29,30].

For variables with a normal distribution, the paired-sample *t*-test was used to compare related groups.

It is essential to highlight that the Saehan dynamometer mechanically records the peak force exerted during the test using a peak-hold needle. By comparing this mechanically recorded maximum force (denoted as mHGS) with the maximum force calculated from the needle’s angular variation captured through video (denoted as vHGS), we can evaluate the reliability of the new testing protocol. We employed Kendall’s tau correlation coefficient to assess the consistency between these two measurements, providing insights into the accuracy and validity of the video-based force measurement compared to the traditional mechanical method. Additionally, a bivariate correlation analysis was conducted on pairs of variables extracted from the sequential recordings, including a two-tailed assessment of the statistical significance of the results.

We found it useful to introduce a new parameter called fatigue over time (FT), for both DH and NDH. This parameter is calculated using a formula that takes into account the maximum HGS (vHGS) and the last recorded value (lHGS) from each set of 750 readings taken at intervals of 33.33 ms, as follows:

FT = (vHGS – lHGS) × 100/vHGS(6)

For all inferential statistical analyses, a statistical significance threshold of *p* ≤ 0.05 was applied. As examples, Poincaré plots representative of a subject were utilized to illustrate the fluctuations in HGS and AV in DH and NDH.

## 3. Results

The results are presented as descriptive statistical metrics in Table 1. The 25-s isometric testing revealed a sharp initial increase in force (with the peak value recorded as vHGS), followed by a gradual decline, accompanied by oscillations in the force values until the end of the test. This pattern is consistent with the expected muscle fatigue dynamics during sustained contractions. The progressive decrease in force likely reflects the fatigue process, while the oscillations could indicate transient adjustments in muscle recruitment aimed at stabilizing force output. These fluctuations may also highlight the involvement of nonlinear physiological mechanisms, which are captured through the Poincaré plot analysis, providing deeper insights into the balance between short-term and long-term variability in HGS.

For both mHGS and vHGS values of the DH and NDH, the Shapiro–Wilk test was conducted, indicating a non-normal distribution of the data. The reliability analysis using Kendall’s tau revealed a significant correlation between mHGS and vHGS, with a coefficient of 0.93 (*p* < 0.001) for the DH and 0.96 (*p* < 0.001) for the NDH. These findings indicate a very strong correlation between the mechanical and video-based measurements, reinforcing the validity of the video processing methodology in HGS assessment.

The next step in the research process refers to the Poincaré analysis. Table 2 presents the mean ± SD values for HGS as well as AV, derived from the data series obtained through video analysis using Kinovea software, version 2023.1.2. The Poincaré plot indices SD_1_, SD_2_, SD_1_/SD_2_, and AFE were computed for each variable, with the Shapiro–Wilk test confirming a non-normal distribution for all.

The results of the Mann–Whitney U test (Table 2) for HGS reveal that the DH demonstrates a trend toward greater long-term variability (SD_2_) and a larger adjustment variability area (AFE) compared to the NDH. Additionally, the mean values of HGS for the tested 25-s interval are approximately 4.86% higher in the case of the DH compared to the NDH. This suggests that the DH exhibits more complex and adaptive motor control strategies, potentially reflecting a higher degree of non-linear variability in force production. Non-linear variability in force suggests a superior capacity to adjust to external conditions, indicating greater efficiency in movement execution and maintaining muscular stability.

In terms of AV, the DH exhibits a value that is approximately 119.84% higher than that of the NDH. Additionally, the DH shows slightly higher short-term variability, as indicated by a higher SD_1_, while SD_2_ remains similar for both hands. The AFE is also marginally larger for the DH than the NDH, reinforcing the idea that the DH exhibits a greater overall variability.

The comparison of Poincaré parameters between the DH and NDH revealed no statistically significant differences across all variables (*p* > 0.05) and effect sizes indicating weak effects. These results suggest that performance between the hands is comparable in the studied population, reflecting potential similarities in daily hand usage and individual variability. Regarding the FT values (Table 1), we observed a normal distribution of the data, with similar mean values between the two hands. By applying the paired-sample *t*-test, no significant differences were found between the means recorded for the DH and NDH.

**Figure 2:**
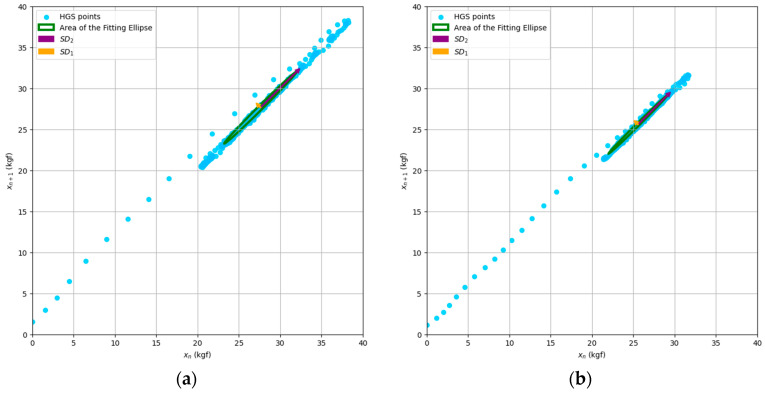
Typical Poincaré plot for a subject’s handgrip strength data: (**a**) dominant hand; (**b**) nondominant hand.

**Figure 3:**
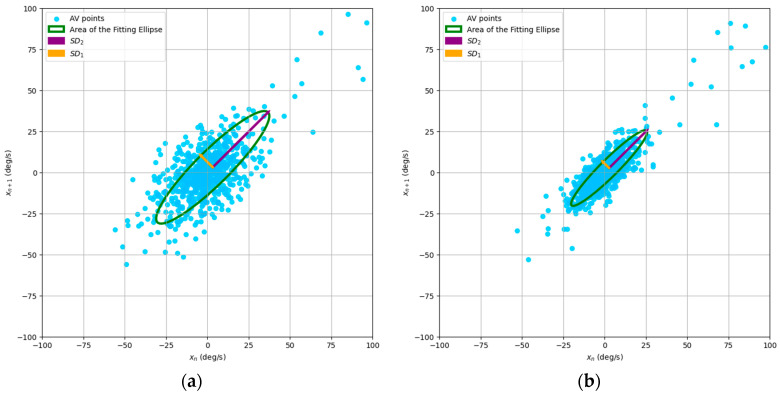
Typical Poincaré plot for a subject’s angular velocity data: (**a**) dominant hand; (**b**) nondominant hand.

From Figure 2 and Figure 3, we can observe typical Poincaré plot representations of the nonlinear variability of HGS and AV, comparing the DH and NDH for a selected subject within the studied group.

The final analysis focused on bivariate non-parametric correlation coefficients (Kendall’s tau) between variables from the recordings, resulting in four correlation matrices for the Poincaré indices of HGS and AV for both DH and NDH (Table 3, Table 4, Table 5 and Table 6). Additionally, we incorporated the variables vHGS and FT into the analysis to evaluate their potential influence on the relationship between grip strength dynamics and measures of variability.

Overall, this correlational analysis reveals significant and specific relationships among HGS, AV, Poincaré indices, and fatigue levels in both DH and NDH, which have implications for understanding hand function and performance.

## 4. Discussion

This study demonstrates the significance of integrating video processing and nonlinear dynamic analysis for assessing HGS variability. We established a strong correlation between mechanical and video-based measurements (*p* < 0.001), validating video assessment as a reliable non-invasive clinical tool. Our analysis revealed significant relationships among HGS, AV, Poincaré indices, and fatigue levels in both DH and NDH (*p* ≤ 0.001). Specifically, higher maximum HGS and AV correlate with increased nonlinear variability in force production.

Our research introduces an innovative approach to analyzing HGS variability by integrating advanced video processing techniques with nonlinear dynamic analysis, specifically through the use of the Poincaré plot method. Unlike traditional methods that focus primarily on peak isometric force, our approach captures the complexity of HGS fluctuations over time, offering a deeper understanding of both short-term and long-term variability. This novel methodology allows for a more precise and dynamic assessment of neuromuscular function, highlighting the differences between the DH and NDH. Additionally, by employing video processing to track force variations and AV, we provide a non-invasive reliable alternative to mechanical measurements, expanding the potential for clinical applications in neuromuscular evaluation and rehabilitation. The study’s findings emphasize the value of nonlinear analysis in uncovering hidden dynamics within physiological data, potentially transforming how HGS is measured and interpreted in both clinical and research settings.

In this context, we start by reporting mHGS and vHGS values in relation to normative data, as shown in Table 1, noting that the means align with expected ranges based on sex and hand laterality for the age group [31,32]. Additionally, both methods yielded slightly higher average values of maximal HGS for the DH compared to the NDH, with an average increase of 5.29% for mHGS and 5.65% for vHGS. However, there were individual cases where the maximum HGS values were greater in the NDH than in the DH. This situation can be explained by the peculiarities of the testing method (isometric contraction with the forearm fixed for 25 s) or the more frequent use of the NDH in everyday activities. Moreover, other authors have reported the less common possibility that the stronger hand may be the nondominant one. This is particularly true for left-handed individuals, who often have to use their right hand out of necessity in physical activities [16]. It should be noted that in the group of subjects, only one was left-handed (3.33%). This percentage is lower than the statistical data regarding the general population [33], but it does not significantly affect the overall results due to the small sample size and the specific characteristics of the testing protocol.

The first notable result to be discussed pertains to the significant correlation observed between the mHGS and vHGS measurements for both the DH and NDH. The strong Kendall’s tau correlation coefficients of 0.93 for the DH and 0.96 for the NDH (*p* < 0.001) demonstrate a high degree of reliability in the video processing method used to assess HGS. The strong agreement between these two methods suggests that video-based assessments can serve as a non-invasive and accurate alternative in clinical settings. This approach not only simplifies data collection but also allows for more detailed dynamic analysis of muscular function over time, offering enhanced diagnostic capabilities for evaluating neuromuscular health and fatigue. Therefore, the clinical utility of this method lies in its ability to provide reliable measurements while enabling deeper insights into muscle performance and variability, particularly useful in rehabilitation and functional assessments.

The applicability of this new method is evident, especially considering HGS is emerging as a proposed new vital sign and key biomarker of health, strongly linked to physical capability and sarcopenia, and predictive of morbidity and mortality, particularly in aging populations [4]. While numerous HGS evaluation protocols have been proposed [34], emerging technologies such as digital handgrip dynamometry and accelerometry offer novel ways to assess muscle function more comprehensively [35]. Other researchers have also explored advanced methods that go beyond traditional strength measurements, providing insights into force variability, fatigability, and motor control [36]. These approaches align with the objectives of this study, as the integration of video processing and nonlinear analysis similarly enhances the understanding of muscle dynamics and variability, offering valuable clinical and research applications.

In the context of our experimental design, the dynamic patterns observed during the 25-s isometric test, comprising 750 recorded data points at 33.33 ms intervals, facilitated a comprehensive nonlinear analysis of HGS variability. The recorded data illustrated a typical progression: an initial sharp increase in force leading to a peak value (vHGS), followed by a plateau characterized by oscillations and a subsequent gradual decline indicative of muscle fatigue. These oscillations likely represent the body’s compensatory adjustments in muscle recruitment aimed at stabilizing force output, while the overall decline corresponds to established fatigue dynamics. The introduction of the new parameter, FT, which quantifies the reduction in force from the peak to the final value, offers a more nuanced understanding of the fatigue process during sustained contractions. This nonlinear approach, achieved through high-frequency sampling, yields significant insights into both short-term and long-term variability in muscle performance, thereby enriching the interpretation of HGS data and reinforcing the clinical relevance of our findings.

To model the nonlinear dynamics of HGS and AV, we used phase space reconstruction through the Poincaré plot, mapping force and angular velocity measurements onto points in a Cartesian plane. This approach revealed muscle recruitment patterns, capturing both short-term fluctuations (SD_1_) and long-term variability (SD_2_). The AFE quantified overall fluctuations, reflecting the system’s adaptability. These indices provided insights into muscle stability and adjustment during fatigue. The nonlinear dynamics are visualized in the examples shown in Figure 1 and Figure 2.

Our study is consistent with the emerging trend of exploring new indicators derived from the force–time curve to enhance muscle function assessment. Similar to proposed metrics such as grip fatigue and fatigue resistance [37], our methodology emphasizes capturing detailed dynamics in HGS. Additionally, the nonlinear nature of the extracted data makes it well-suited for Poincaré analysis, offering advantages over traditional linear methods. While linear analysis assumes direct relationships between variables, nonlinear analysis captures the complex interactions and variations typical of physiological systems [38]. This approach can reveal patterns that linear methods may overlook, leading to a more comprehensive understanding of muscle dynamics. Furthermore, nonlinear techniques often connect with the concept of entropy, quantifying the degree of unpredictability or disorder within the system [39,40].

Another novel aspect introduced in the data analysis is AV, derived from Kinovea software, version 2023.1.2. In contrast to the traditional force measurement during dynamic isometric testing, AV provides critical insights into the rate of force development and the muscle’s contraction dynamics. This metric enhances our understanding of muscle physiology by revealing how quickly force is generated, which is essential for evaluating muscle recruitment patterns and coordination. Other authors have validated force–velocity assessments as feasible, valid, and cost-effective alternatives to isokinetic dynamometry for evaluating muscle function in healthy older adults [41]; the inclusion of AV enhances this assessment. This is physiologically significant, as it reflects the efficiency of motor unit recruitment and synchronization, critical for optimal muscle performance [42]. Furthermore, measuring AV helps assess how quickly a muscle generates force relative to its capacity, aiding in understanding fatigue mechanisms and overall muscle functionality.

The Poincaré analysis of force dynamics in our study indicates that the DH exhibits greater long-term variability (SD_2_) and a larger adjustment variability area (AFE) compared to the NDH, suggesting a more complex motor control strategy, though these differences are not statistically significant. Regarding AV, the DH demonstrates slightly greater short-term variability, as indicated by a higher SD_1_. Despite these trends, the lack of statistically significant differences suggests that overall performance between the hands remains comparable, likely reflecting similar daily usage patterns.

In our investigation of hand dominance through Poincaré plot analysis, we uncovered subtle differences between the DH and the NDH that reflected functional asymmetry and neuromuscular adaptation. Given that the development of laterality is a dynamic process shaped by both genetic and environmental influences [43], our findings suggest a model of ambidexterity among our group of young adults. This ambidextrous tendency may stem from the frequent use of both hands in daily activities, enhancing bilateral coordination and muscle strength. Neurophysiologically, this can be attributed to the brain’s plasticity, where increased engagement of the NDH strengthens neural pathways. Moreover, minimal practice can significantly improve NDH control in healthy adults by enhancing connectivity between sensorimotor areas and a left-lateralized parieto-prefrontal network [44].

Nevertheless, the static position with the forearm resting on the table may explain the relatively similar values of HGS and the corresponding patterns of variability in both the DH and NDH. It is well-established that handgrip force can vary among individuals depending on anatomical position [45]. It is possible that ambidexterity may become more apparent during a prolonged isometric test lasting 25 s compared to traditional HGS measurements. The continuous muscle engagement required in such an extended task highlights the functional abilities of both hands, allowing for a deeper assessment of their endurance and motor control. As the muscles fatigue (FT), differences between the DH and NDH may emerge, revealing how ambidextrous individuals adapt their strategies to maintain performance over time. This sustained effort could also promote neuromuscular adaptations, particularly in the NDH, thereby enhancing coordination and strength. Overall, the complexity of a prolonged isometric task provides a richer context for evaluating bilateral coordination and may better illustrate the nuances of ambidexterity than traditional strength assessments. This aligns with the principle suggested by some authors regarding the difference between hand preference and hand performance [46].

From the same perspective, we did not find significant differences in FT between the DH and NDH in the applied test. Other authors have noted inconsistencies in the literature regarding the relationship between endurance and laterality during a 30-s isometric handgrip strength (HGS) test, measured with an electronic dynamometer. In this context, endurance was defined as the ratio between the forces measured during the first and last seconds [47].

A final discussion addresses the results of the correlational analysis, which revealed several statistically significant correlations of interest. In Table 3, in the DH, we observe a strong correlation between vHGS and HGS (τ = 0.71, *p* ≤ 0.001), along with a moderate correlation between vHGS and AFE (τ = 0.64, *p* ≤ 0.001) and a moderate correlation between vHGS and SD2 (τ = 0.56, *p* ≤ 0.001). These results indicate that higher maximum grip strength is associated with greater mean overall grip strength over 25 s and variability patterns (AFE and SD2) in DH.

For the NDH, as shown in Table 4, there is a strong correlation between vHGS and HGS (τ = 0.76, *p* ≤ 0.001), a strong correlation between vHGS and AFE (τ = 0.76, *p* ≤ 0.001), and a moderate correlation between vHGS and SD2 (τ = 0.63, *p* ≤ 0.001). Additionally, a moderate correlation exists between vHGS and SD1 (τ = 0.52, *p* ≤ 0.001). These findings suggest that maximum grip strength is linked to variability patterns as measured by AFE, SD2, and SD1 in the NDH.

In the analysis of AV in the DH, as shown in Table 5, a moderate correlation exists between vHGS and AV (τ = 0.63, *p* ≤ 0.001), indicating that greater grip strength is associated with higher AV. Additionally, a moderate negative correlation is observed between AV and FT (τ = −0.3, *p* ≤ 0.019), suggesting that increased AV may be associated with reduced fatigue. Weak correlations are noted between vHGS and SD2 (τ = 0.25, *p* ≤ 0.05) and between vHGS and AFE (τ = 0.25, *p* ≤ 0.05. Overall, these findings underscore the interconnectedness of grip strength dynamics, AV, and variability patterns in DH function.

The last analysis of the correlations for AV in the NDH, as shown in Table 6, brings into focus that a moderate correlation exists between vHGS and AFE (τ = 0.57, *p* ≤ 0.001). This is followed by moderate correlations between vHGS and SD1 (τ = 0.56, *p* ≤ 0.001) and between vHGS and SD2 (τ = 0.53, *p* ≤ 0.001). A moderate correlation is also observed between vHGS and AV (τ = 0.44, *p* ≤ 0.001), showing that a stronger grip is linked to elevated AV. Additionally, a moderate negative correlation is noted between AV and FT (τ = −0.44, *p* ≤ 0.001), suggesting that a higher AV may correlate with reduced fatigue. These results elucidate the complex interrelationships among grip strength, AV, variability metrics, and fatigue in the NDH.

The analysis reveals significant relationships between HGS, AV, Poincaré indices, and fatigue levels in both DH and NDH (*p* ≤ 0.001). Nonlinear variability, as demonstrated by the Poincaré indices, shows important correlations with maximum HGS (vHGS), regardless of hand dominance, with some particularities when ranking Kendall’s tau coefficients. Additionally, significant correlations were found between the Poincaré indices and AV, although with lower tau values for both hands. It can be suggested that individuals with greater maximum HGS may exhibit higher nonlinear variability in force production, which could imply a form of adaptable motor control that allows them to maintain performance under varying conditions. However, this increased force and AV might come at the expense of greater variability, raising questions about the balance between strength, dynamic force production, and the stability of these variables. Thus, while faster and more dynamic movements are often associated with effective muscle recruitment and coordination, the relationship between variability and performance remains complex and not entirely straightforward. Nonetheless, a significant portion of the debate surrounding human movement variability in the literature can be attributed to the methodologies employed in various studies [48].

On the other hand, moderate negative correlations emerged between AV and FT for both hands, indicating that higher AV and faster force variation are associated with reduced fatigue. Physiologically, this relationship suggests that muscles capable of generating faster force variation (higher AV) may recruit motor units more efficiently, reducing the overall fatigue experienced during sustained contractions. This can be further explained by the property of motor unit synchronization, where the relative timing of action potential discharges among motor units differs across contraction types. Greater synchronization during rapid force generation may enhance motor unit efficiency, allowing for more sustained force output with less fatigue [49].

The strength of this study lies in its innovative approach to analyzing HGS variability by integrating dynamometry and advanced video processing techniques with nonlinear dynamic analysis through the Poincaré plot method. This approach provides a comprehensive understanding of the complexities in HGS fluctuations over time, allowing for a more precise assessment of neuromuscular function. The findings underscore the reliability of video-based assessments as a non-invasive alternative to traditional mechanical measurements, which can enhance diagnostic capabilities in clinical settings. By capturing both short-term and long-term variability, this study contributes valuable insights into muscle dynamics, functional capacity, and the implications of hand dominance.

Future research should adopt an ontogenetic perspective to explore how the relationships between HGS and nonlinear variability develop across the lifespan. Investigating how these factors evolve from childhood through adulthood can provide insights into the impact of training, motor learning, and developmental stages on muscle performance. Additionally, understanding how these dynamics differ in various populations (such as athletes versus non-athletes or healthy individuals versus those with neuromuscular conditions) could inform targeted interventions and rehabilitation strategies. This ontogenetic approach may also reveal critical periods for enhancing neuromuscular function and adaptability, ultimately contributing to improved health and performance throughout life.

Our study has several limitations to consider. The sample size of 30 participants may restrict the generalizability of the findings. Additionally, focusing on healthy young adults aged 20 to 24 years may limit applicability to older populations or those with neuromuscular conditions. Errors related to video processing could also impact accuracy as any movement of the hand and dynamometer during isometric testing might distort the angle measurement and subsequently affect the calculated force output. Furthermore, self-reported hand dominance may introduce bias, as some participants could have ambidextrous tendencies that were not captured. Future research should involve larger more diverse samples and address these potential measurement errors to enhance reliability.

## 5. Conclusions

This study emphasizes the integration of advanced video processing and nonlinear dynamic analysis for assessing HGS variability. We confirmed a strong correlation between mechanical and video-based measurements, supporting the video assessment as a reliable non-invasive method in clinical settings. The results demonstrate the utility of nonlinear analysis in revealing the dynamics of neuromuscular function and fatigue. Significant correlations among HGS, AV, Poincaré indices, and fatigue levels in both DH and NDH indicate that higher maximum HGS and AV are associated with increased nonlinear variability in force production. This approach improves neuromuscular assessment and opens new avenues for HGS research in diverse populations.

## Figures and Tables

**Table 1:** Summary of the characteristics of study participants (n = 30).

Variable	Age (Years)	mHGS DH (kgf)	vHGS DH (kgf)	mHGS NDH (kgf)	vHGS NDH (kgf)	FT DH %	FT NDH %
mean	21.6	34.25	35.36	32.53	33.47	39.65	39.69
SD	1.3	9.53	9.26	11.11	10.83	10.25	16.88
minimum	20	22.00	23.43	16.50	18.21	25.84	42.52
maximum	24	64.50	65.24	61.50	62.86	16.90	10.28

Note—mHGS: Maximum handgrip strength measured mechanically using a dynamometer; vHGS: maximum handgrip strength assessed through video recording; FT: fatigue over time; DH: dominant hand: NDH: nondominant hand; SD: standard deviation; n: number of subjects.

**Table 2:** Comparison of recorded Poincaré parameters between the dominant hand and nondominant hand in subjects (n = 30) using the Mann–Whitney U test.

Parameter	DH Mean ± SD	NDH Mean ± SD	U	Z	*p*	r
vHGS	35.36 ± 9.26	33.47 ± 10.83	371	−1.17	0.24	−0.21
HGS	26.11 ± 7.56	24.90 ± 9.11	385	−0.96	0.34	−0.18
SD_1_	0.24 ± 0.08	0.24 ± 0.08	434.5	−0.23	0.82	−0.04
SD_2_	7.01 ± 2.45	6.28 ± 2.83	343	−1.58	0.11	−0.29
SD_1_/SD_2_	0.04 ± 0.01	0.04 ± 0.02	406.5	−0.66	0.51	−0.12
AFE	5.55 ± 3.57	5.07 ± 3.95	364	−1.27	0.20	−0.23
AV	5.53 ± 15.32	2.52 ± 1.06	355.5	−1.40	0.16	−0.26
SD_1_	9.01 ± 5.65	8.67 ± 5.33	414.5	−0.53	0.6	−0.01
SD_2_	43.97 ± 13.57	43.95 ± 14.57	444.5	−0.08	0.94	−0.01
SD_1_/SD_2_	0.20 ± 0.09	0.19 ± 0.07	428.5	−0.32	0.75	−0.05
AFE	1408.87 ± 1360.91	1402.87 ± 1433.61	423	−0.40	0.69	−0.07

Note—vHGS: maximum handgrip strength assessed through video recording; HGS: hand grip strength; AV: angular velocity; DH: dominant hand: NDH: nondominant hand; SD: standard deviation; AFE area of the fitting ellipse; U: Mann–Whitney’s U-test values; z: scores; *p*: thresholds of statistical significance; r: effect size coefficients; n: number of subjects.

**Table 3:** Bivariate correlation matrix (Kendall’s tau) for handgrip strength in dominant hand (n = 30).

Variable		vHGS	HGS	SD_1_	SD_2_	SD_1_/SD_2_	AFE	FT
vHGS	τ	1	0.71 *	0.28 *	0.56 *	−0.23	0.64 *	−0.01
	*p*		0.001	0.035	0.000	0.103	0.001	0.97
HGS	τ	0.71 *	1	0.25	0.43 *	−0.16	0.52 *	−0.21
	*p*	0.001		0.055	0.001	0.263	0.001	0.11

Note—vHGS: maximum handgrip strength assessed through video recording; HGS: hand grip strength; SD: standard deviation; AFE area of the fitting ellipse; FT: fatigue over time; n: number of subjects, τ: Kendall’s tau correlation coefficient; *p*: significance level; * correlation is significant (two-tailed).

**Table 4:** Bivariate correlation matrix (Kendall’s Tau) for handgrip strength in the nondominant hand.

Variable		vHGS	HGS	SD_1_	SD_2_	SD_1_/SD_2_	AFE	FT
vHGS	τ	1	0.76 *	0.52 *	0.63 *	−0.18	0.76 *	0.1
	*p*		0.001	0.001	0.001	0.196	0.001	0.422
HGS	τ	0.76 *	1	0.59 *	0.41 *	0.03	0.6 *	−0.06
	*p*	0.001		0.001	0.001	0.810	0.001	0.643

Note—vHGS: maximum handgrip strength assessed through video recording; HGS: hand grip strength; SD: standard deviation; AFE area of the fitting ellipse; FT: fatigue over time; n: number of subjects; τ: Kendall’s tau correlation coefficient; *p*: significance level; * correlation is significant (two-tailed).

**Table 5:** Bivariate correlation matrix (Kendall’s Tau) for angular velocity in the dominant hand.

Variable		vHGS	AV	SD_1_	SD_2_	SD_1_/SD_2_	AFE	FT
vHGS	τ	1	0.63 *	0.22	0.25 *	0.15	0.25 *	−0.01
	*p*		0.001	0.087	0.050	0.267	0.050	0.972
AV	τ	0.63 *	1	0.15	0.17	0.13	0.14	−0.3 *
	*p*	0.001		0.239	0.187	0.316	0.284	0.019

Note—vHGS: maximum handgrip strength assessed through video recording; AV: angular velocity; SD: standard deviation; AFE area of the fitting ellipse; FT: fatigue over time; n: number of subjects; τ: Kendall’s tau correlation coefficient; *p*: significance level; * correlation is significant (two-tailed).

**Table 6:** Bivariate correlation matrix (Kendall’s Tau) for angular velocity in the nondominant hand.

Variable		vHGS	AV	SD_1_	SD_2_	SD_1_/SD_2_	AFE	FT
vHGS	τ	1	0.44 *	0.56 *	0.53 *	0.36	0.57 *	0.1
	*p*		0.001	0.001	0.001	0.006	0.001	0.422
AV	τ	0.44 *	1	0.31 *	0.33 *	0.17	0.33 *	−0.44 *
	*p*	0.001		0.017	0.010	0.197	0.010	0.001

Note—vHGS: maximum handgrip strength assessed through video recording; AV: angular velocity; SD: standard deviation; AFE area of the fitting ellipse; FT: fatigue over time; n: number of subjects; τ: Kendall’s tau correlation coefficient; *p*: significance level; * correlation is significant (two-tailed).

## Data Availability

The data are available on request from the corresponding author. All data relevant to the study are included in the article.
